# Subcutaneous Endosalpingiosis of the Buttock Without Prior Surgery or Trauma: A Case Report and Literature Review

**DOI:** 10.7759/cureus.91316

**Published:** 2025-08-30

**Authors:** Rui Suzuki

**Affiliations:** 1 Plastic and Reconstructive Surgery, Beppu Medical Center, Beppu, JPN

**Keywords:** buttock mass, endosalpingiosis, immunohistochemistry, müllerianosis, subcutaneous tumor

## Abstract

Endosalpingiosis is a rare, benign condition characterized by the presence of ectopic glandular epithelium that resembles the epithelium of the fallopian tubes. It typically affects the pelvic peritoneum and is often discovered incidentally. We present a rare case of subcutaneous endosalpingiosis localized in the left buttock of a 49-year-old woman with no history of surgery or trauma in the area. The patient presented with a palpable, painless subcutaneous mass that had been present for over 15 years and caused discomfort while sitting. Imaging and aspiration suggested a cystic lesion, and surgical excision was performed under local anesthesia. Histopathological examination confirmed the diagnosis of endosalpingiosis confined to the subcutaneous tissue. This report highlights the clinical and pathological features of this extremely rare presentation and discusses possible pathogenesis with reference to previously published literature.

## Introduction

Endosalpingiosis is a benign condition characterized by the ectopic presence of fallopian tube-like ciliated epithelium. It is most frequently found on the pelvic peritoneum, ovaries, and fallopian tubes and is considered part of the Müllerianosis spectrum, along with endometriosis and endocervicosis [1-3]. While usually discovered incidentally during pelvic surgeries, extrapelvic presentations are exceedingly rare. Subcutaneous involvement, particularly in the buttocks, is exceptionally rare and presents a significant diagnostic challenge.

## Case presentation

**Figure 1:**
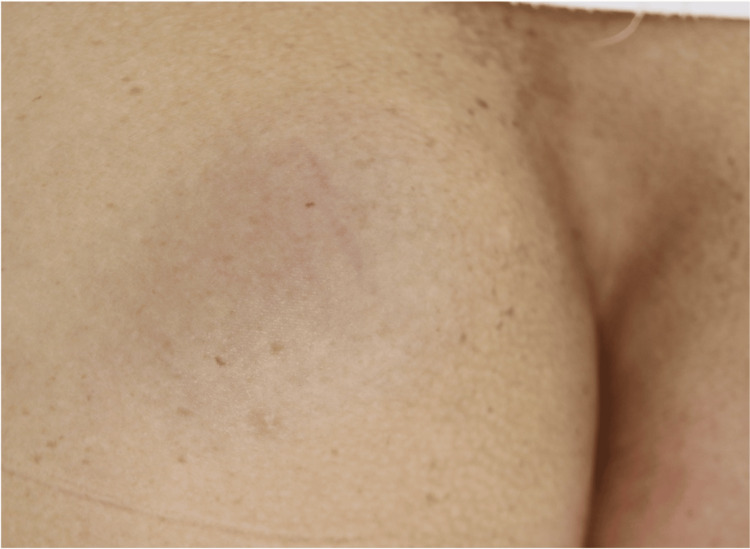
Clinical Findings A subcutaneous tumor approximately 6 cm in size was noted on the left buttock, with a pale bluish discoloration of the overlying skin.

A 49-year-old woman was referred to our department for evaluation of a left buttock subcutaneous mass that was first noticed approximately 15 years prior. She reported no associated pain but experienced discomfort while sitting. Her medical history was notable for Graves' disease. She denied any history of trauma or prior surgical procedures in the buttock region. The physical examination revealed a soft, well-circumscribed, and mobile 6-cm subcutaneous nodule on the left buttock (Figure 1).

**Figure 2:**
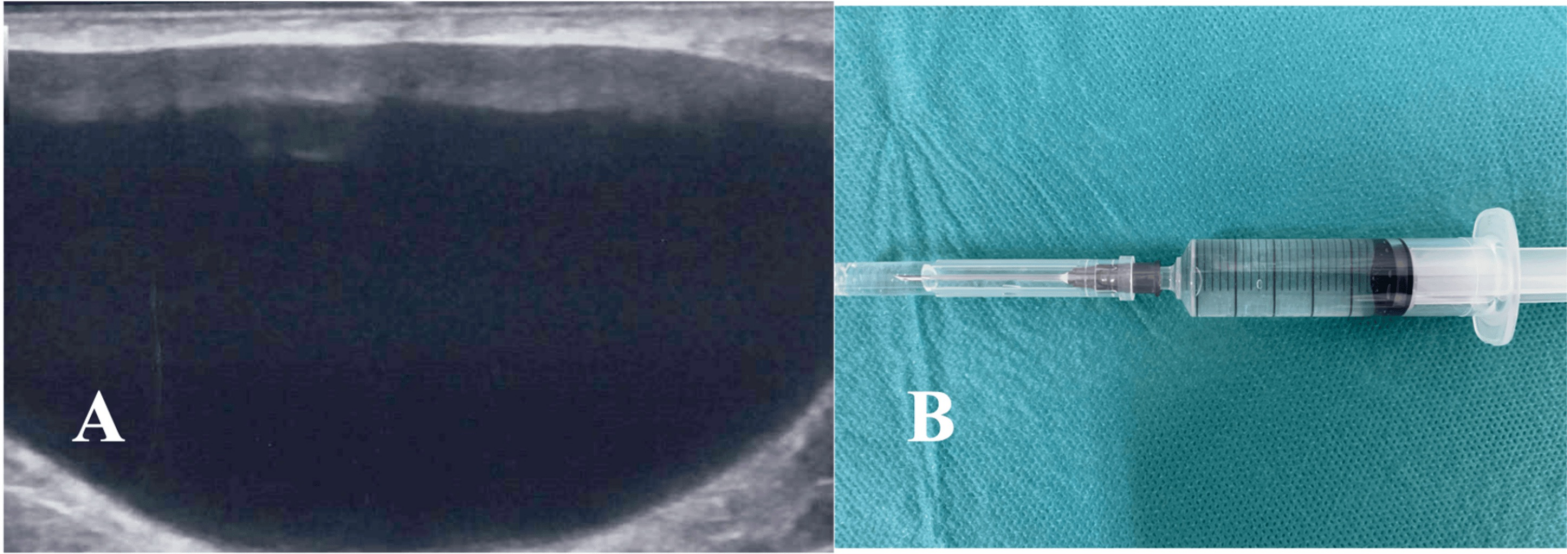
Imaging and Cytology (A) Ultrasonography reveals a cystic lesion beneath the dermis. (B) Fine-needle aspiration yields clear, serous fluid.

Ultrasonography demonstrated a round, hypoechoic lesion without internal vascularity. Fine-needle aspiration yielded clear, serous fluid (Figure 2).

**Figure 3:**
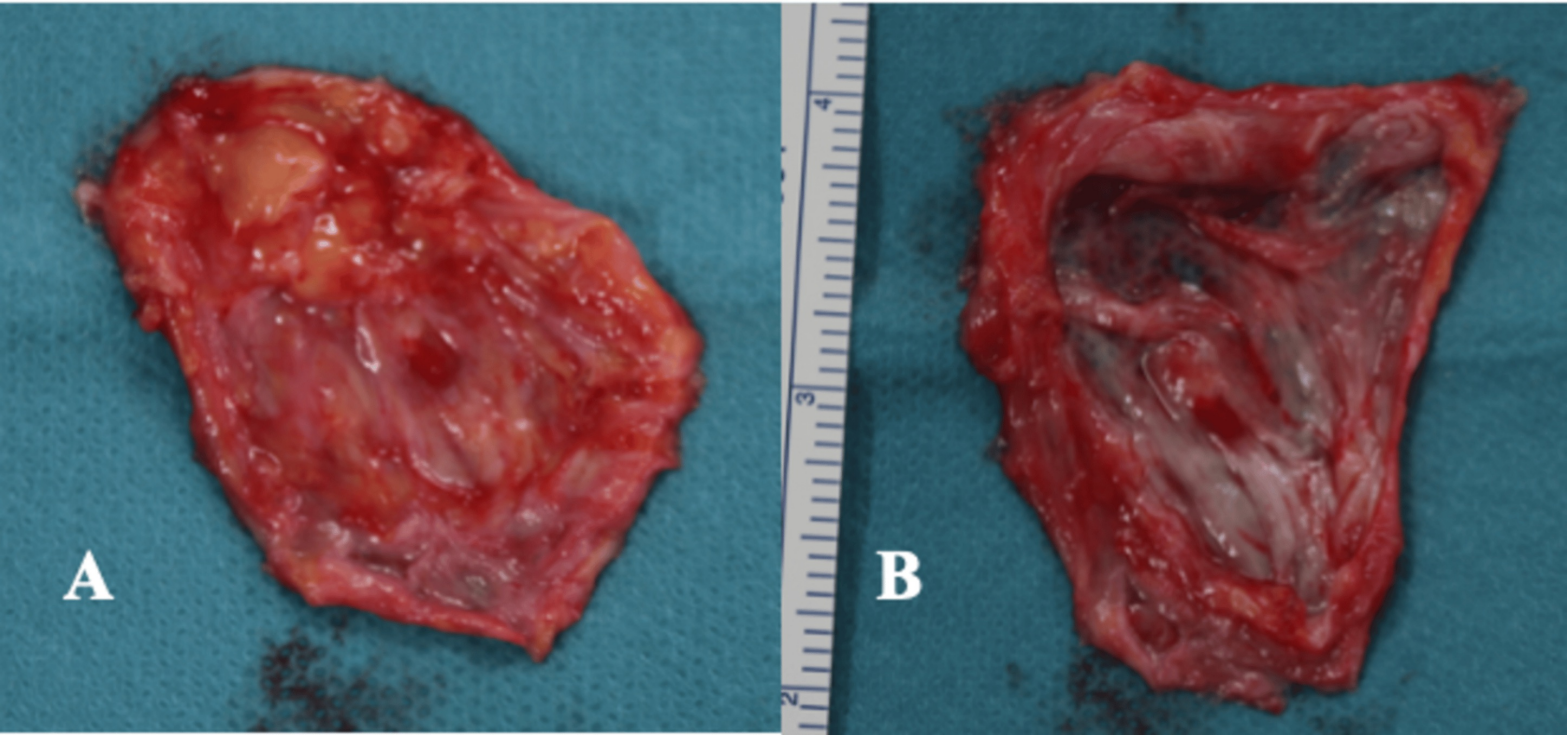
Intraoperative Findings (A, B) A unilocular cystic lesion with a thin capsule measuring slightly over 4 cm was identified. The inner surface was smooth and contained colorless, transparent serous fluid without solid components.

The cyst rapidly reaccumulated fluid following aspiration, prompting surgical excision under local anesthesia. The gross examination showed a well-encapsulated cystic lesion (Figure 3).

**Figure 4:**
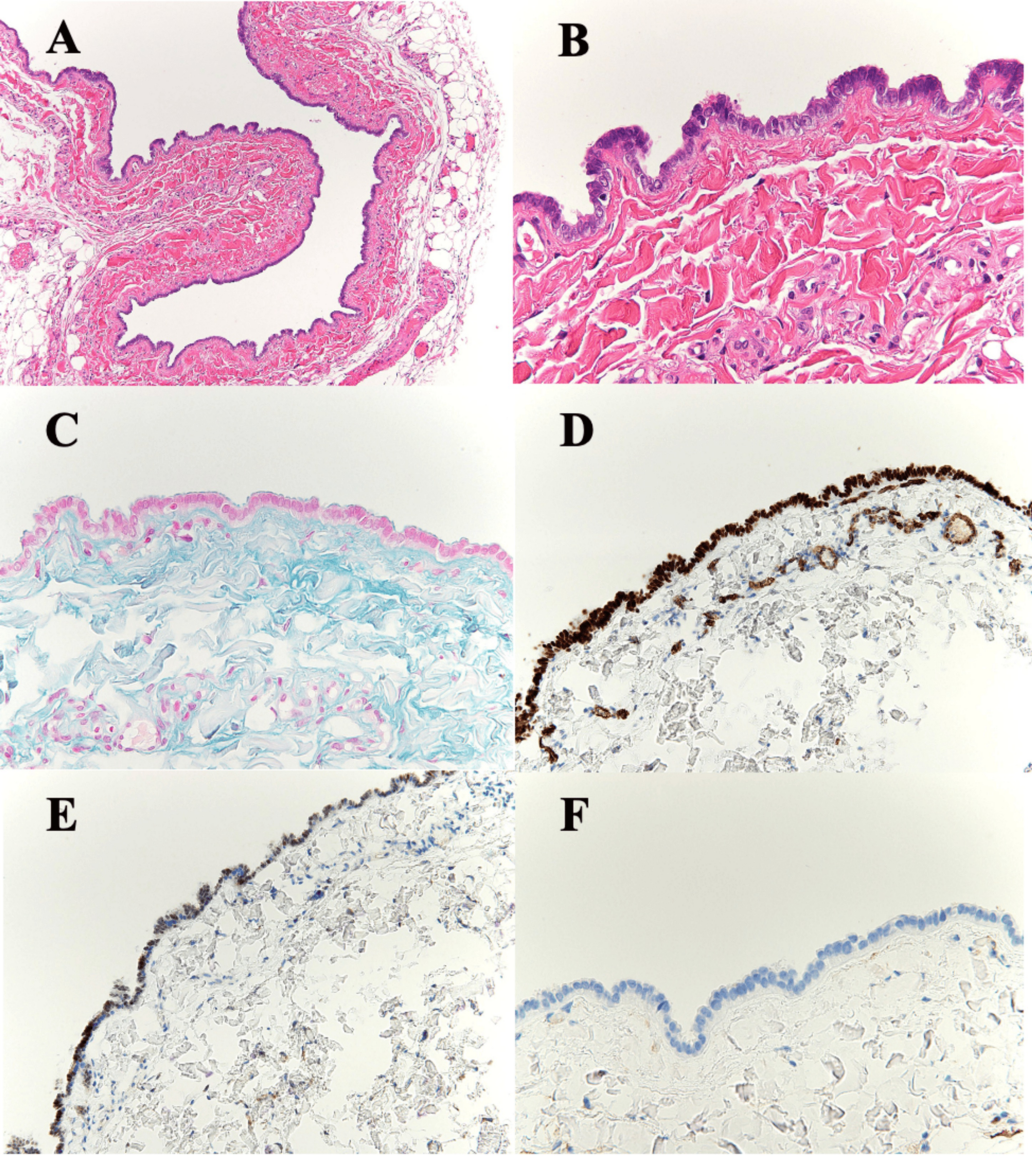
Histopathological Findings (A, B) Hematoxylin and eosin (H&E) staining: The cyst wall is lined with simple cuboidal epithelium exhibiting cilia and no cytological atypia. Focal luminal projections are present. (C) Alcian blue staining: Linear positivity is observed along the brush border of the epithelial cells. (D, E) *PAX8* immunostaining: Approximately half of the epithelial cell nuclei are positive. (F) WT-1 immunostaining: Strong diffuse nuclear positivity is observed.

Histopathological analysis revealed cystic structures lined with benign ciliated tubal-type epithelium. Alcian blue staining revealed linear positive findings on the brush border of the cuboidal epithelial cells. Immunohistochemical staining demonstrated *PAX8 *(paired-box gene 8) positivity in a subset of epithelial cell nuclei, with strong, diffuse WT-1 (Wilms' tumor 1) nuclear positivity (Figure 4). The lesion was confined to the subcutaneous tissue, with no evidence of atypia or malignancy.

## Discussion

Endosalpingiosis was first described by Sampson in 1930 to denote fallopian tube epithelium-like structures observed within scar tissue following salpingectomy [1].

The pathogenesis of endosalpingiosis is presumed to follow mechanisms similar to other forms of Müllerianosis. This group of conditions constitutes the non-neoplastic disorders of the Müllerian system. Two major theories have been proposed to explain the development of endosalpingiosis. One theory posits that endometrial cells or their precursors are transported via various routes (transtubal, hematogenous, lymphatic, or direct implantation) and become established in ectopic locations [4]. The other theory suggests that Müllerian ectopias result either from metaplastic changes in the target organ (the coelomic metaplasia theory or secondary Müllerian system) or from remnants of displaced embryonic Müllerian tissue [5]. We consider metaplastic processes or embryonic Müllerian rests to be more plausible explanations in this instance.

Non-neoplastic glandular proliferations showing spontaneous Müllerian differentiation have been described in various sites, including the vagina, uterine cervix, urinary bladder, appendix, peritoneum, abdominal wall (inguinal canal, umbilicus), and lymph nodes [4,6-13]. However, to the best of our knowledge, this is the first report of spontaneous endosalpingiosis presenting as a subcutaneous nodule in the buttock.

Endosalpingiosis is influenced by hormonal status and, similar to endometriosis, is most frequently observed in premenopausal women. However, it has also been reported in postmenopausal women [14] and even in men [15]. Although few studies have summarized its prevalence, Zinsser et al. reported endosalpingiosis in 13 of 128 patients (10.3%) who underwent gynecologic surgery, and Tohya et al. identified it in three of 18 cases (16.7%) who underwent peritoneal biopsy for endometriosis [11,16].

Clinically, although endosalpingiosis may occur in conjunction with hormonal cycles, it differs clinically from endometriosis. It is not typically associated with chronic pelvic pain, dysmenorrhea, or infertility [16]. In our case, the patient was asymptomatic apart from palpable subcutaneous swelling, and there were no periodic changes in tumor size. This is likely due to the nature of tubal epithelium, which does not exhibit the cyclical proliferative and inflammatory behavior seen in endometrial tissue.

Histologically, endosalpingiosis is characterized by cystic or glandular structures lined by a single layer of benign cuboidal to columnar epithelium. To confirm the Müllerian origin of the lesion, immunohistochemical staining is essential. Various specific markers have been developed to narrow down the tissue of origin. In our case, we utilized *PAX8* and WT-1 to confirm Müllerian differentiation. *PAX8* encodes a transcription factor associated with embryogenesis and tumor development, and its expression has been confirmed in the thyroid, kidney, brain, and Müllerian-derived tissues, including the fallopian tubes [17]. WT-1, which is crucial for the development of the urogenital tract and mesothelium, is known to be regulated by *PAX8* expression. These markers are now widely used in the diagnosis of ovarian neoplasms [18]. In our case, immunohistochemical staining for CD10, a marker of endometrial stromal tissue [19], was negative, thereby supporting a diagnosis of endosalpingiosis of fallopian tube origin rather than endometriosis. As mentioned, endosalpingiosis may arise incidentally in various anatomical locations, often in atypical sites. In such cases, the diagnosis can be challenging. When a tumor shows glandular architecture histologically, Müllerian-origin tumors should be considered in the differential diagnosis. It is important to include immunostaining for Müllerian markers, such as *PAX8* and WT-1, as well as common markers like estrogen receptor (ER) and progesterone receptor (PR), to aid in accurate diagnosis.

Regarding treatment, unlike endometriosis, endosalpingiosis is not typically associated with cyclic pain or tumor growth in response to hormonal fluctuations. Consequently, hormone-based therapies such as oral contraceptives or GnRH agonists are generally considered ineffective [11]. Therefore, in the absence of symptoms, conservative management with observation may be appropriate. However, in clinical practice, especially for superficial cystic lesions, surgical excision, including the cyst wall, is preferable for both diagnosis and treatment due to the difficulty of making a definitive clinical diagnosis.

## Conclusions

We report an extremely rare case of subcutaneous endosalpingiosis of the buttock in a patient with no surgical or traumatic history. This case expands the known clinical spectrum of endosalpingiosis and underscores the importance of considering it in the differential diagnosis of cystic subcutaneous lesions, even in atypical locations.

## References

[1] Sampson JA (1930). Postsalpingectomy endometriosis (endosalpingiosis). Am J Obstet Gynecol.

[2] Clement PB (1987). Endometriosis, lesions of the secondary Müllerian system, and pelvic mesothelial proliferations. Blaustein's Pathology of the Female Genital Tract.

[3] Papavramidis TS, Sapalidis K, Michalopoulos N, Karayannopoulou G, Cheva A, Papavramidis ST (2010). Umbilical endosalpingiosis: a case report. J Med Case Rep.

[4] Lauchlan SC (1972). The secondary Müllerian system. Obstet Gynecol Surv.

[5] Sampson JA (1940). The development of the implantation theory for the origin of peritoneal endometriosis. Am J Obstet Gynecol.

[6] Martinka M, Allaire C, Clement PB (1999). Endocervicosis presenting as a painful vaginal mass: a case report. Int J Gynecol Pathol.

[7] Young RH, Clement PB (2000). Endocervicosis involving the uterine cervix: a report of four cases of a benign process that may be confused with deeply invasive endocervical adenocarcinoma. Int J Gynecol Pathol.

[8] Nazeer T, Ro JY, Tornos C, Ordonez NG, Ayala AG (1996). Endocervical-type glands in urinary bladder: a clinicopathologic study of six cases. Hum Pathol.

[9] Clement PB, Young RH (1992). Endocervicosis of the urinary bladder. A report of six cases of a benign Müllerian lesion that may mimic adenocarcinoma. Am J Surg Pathol.

[10] Cajigas A, Axiotis CA (1990). Endosalpingiosis of the vermiform appendix. Int J Gynecol Pathol.

[11] Zinsser KR, Wheeler JE (1982). Endosalpingiosis in the omentum: a study of autopsy and surgical material. Am J Surg Pathol.

[12] Horton JD, Dezee KJ, Ahnfeldt EP, Wagner M (2008). Abdominal wall endometriosis: a surgeon's perspective and review of 445 cases. Am J Surg.

[13] Sinkre P, Hoang MP, Albores-Saavedra J (2002). Mullerianosis of inguinal lymph nodes: report of a case. Int J Gynecol Pathol.

[14] Maeda K, Kojima F, Ishida M, Iwai M, Kagotani A, Kawauchi A (2014). Müllerianosis and endosalpingiosis of the urinary bladder: report of two cases with review of the literature. Int J Clin Exp Pathol.

[15] Gallan AJ, Antic T (2016). Benign müllerian glandular inclusions in men undergoing pelvic lymph node dissection. Hum Pathol.

[16] Tohya T, Nakamura M, Fukumatsu Y, Katabuchi H, Matsuura K, Itoh M, Okamura H (1991). Endosalpingosis in the pelvic peritoneum and pelvic lymph nodes (Article in Japanese). Nihon Sanka Fujinka Gakkai Zasshi.

[17] Munakata S, Yamamoto T (2015). Incidence of serous tubal intraepithelial carcinoma (STIC) by algorithm classification in serous ovarian tumor associated with PAX8 expression in tubal epithelia: a study of single institution in Japan. Int J Gynecol Pathol.

[18] Rhodes A, Vallikkannu N, Jayalakshmi P (2017). Expression of WT1 and PAX8 in the epithelial tumours of Malaysian women with ovarian cancer. Br J Biomed Sci.

[19] Toki T, Shimizu M, Takagi Y, Ashida T, Konishi I (2002). CD10 is a marker for normal and neoplastic endometrial stromal cells. Int J Gynecol Pathol.

